# Liver radiofrequency ablation as emergency treatment for a ruptured hepatocellular carcinoma: a case report

**DOI:** 10.1186/s13256-017-1199-1

**Published:** 2017-03-01

**Authors:** Alessandra Bertacco, Francesco D’Amico, Maurizio Romano, Michele Finotti, Alessandro Vitale, Umberto Cillo

**Affiliations:** 1 Department of Surgery, Oncology and Gastroenterology (DISCOG), Hepatobiliary Surgery and Liver Transplantation, Padua University, Padua, Italy; 2 Department of Surgery, Division of Transplantation and Immunology, Yale University, New Haven, Connecticut USA

**Keywords:** HCC ruptured, Radiofrequency ablation, Liver bleeding, Case report

## Abstract

**Background:**

Hemoperitoneum is a possible complication of hepatocellular carcinoma that may require emergency surgery as an alternative to radiological locoregional therapies.

**Case presentation:**

We present a case report of a 78-year-old white man with alcoholic-related cirrhosis and a multifocal hepatocellular carcinoma. An abdominal computed tomography scan showed multiple and bilateral foci of bleeding from broken liver cancer. He was urgently transferred from our radiology unit to our operating room for massive hemoperitoneum. A middle line laparotomy detected a massive hemoperitoneum. His liver was cirrhotic and completely subverted by a tumor; there were two spontaneous bleeding lacerations on segments II and IV, which were uncontrollable with conventional hemostatic techniques. Therefore, it was decided to carry out the coagulation of the multiple vascular afferents of each single mass by means of radiofrequency ablation cycles performed circumferentially on both nodules for a total of 40 minutes. Hemostasis was achieved; the radiofrequency ablation controlled the bleeding from his ruptured hepatocellular carcinoma. He was transferred to our intensive care unit for postoperative monitoring in terms of hemodynamic stability. On postoperative day 2 he was discharged from our intensive care unit.

**Conclusions:**

Multifocal bleeding hepatocellular carcinoma still has an extremely high mortality. The angiographic control of multiple bilateral bleeding lesions can be extremely difficult and can be contraindicated by the location of the lesions and by the overall clinical condition of the patient. In this case, treatment with radiofrequency ablation has proven to be effective in the control of multiple and bilateral hepatic lesions. This particular technique allowed us to attack the lesion at the level of the vascular pedicle in order to control the bleeding.

## Background

Spontaneous liver bleeding (SLB) is a rare life-threatening complication that warrants a timely diagnosis and effective control of hemorrhage. Hepatocellular carcinoma (HCC), after benign adenoma, is the most frequent underlying pathology of SLB [1]. HCC is the fifth most prevalent cancer in the world. More than 80 % of HCCs develop in cirrhotic livers. Liver resection and liver transplantation (LT) offer the best chance of cure [2]. Spontaneous HCC rupture is not a rare first presentation of the disease and bleeding is one of the most serious complications. While HCC has often been detected precociously in recent years, an incidence of ruptured HCC of 3 to 15 % has been reported and its in-hospital mortality rate ranges from 7 to 25 % in the acute phase [2, 3]. Spontaneous HCC rupture is likely to occur in patients with advanced stage HCC with reported incidences of 10.0 % in Japan [4], 12.4 % in Thailand [5], and approximately 3.0 % in the UK [6]. Patients with ruptures could have favorable long-term outcomes, particularly those without decompensated liver cirrhosis or portal venous thrombosis and who are eligible for curative treatment [7]. The mechanism of spontaneous rupture of HCC remains unclear, but the risk of bleeding can be explained by modification in the feeding vessels of the tumor, venous congestion, and coagulopathy due to underlying cirrhosis [8, 9]. The management of ruptured HCC is composed of two steps: bleeding control and specific tumor treatment usually at a distance from the acute bleeding episode [1]. There is still a debate concerning the best approach in cases of HCC rupture. The treatment includes emergency liver resections, interventional transcatheter arterial embolization (TAE), packing, and even LT, depending on the hemodynamic stability of the patient, tumor stage, and liver function. Open surgery was the main method used to treat HCC rupture from the 1960s to the 1980s [10, 11]. Recently, a survival benefit of TAE has been reported [12, 13]. Radiofrequency ablation (RFA) was described in rare clinical cases or short series [14], and therefore has not been validated. The theoretical advantage is to treat the underlying tumor if the size is compatible with current indications.

## Case presentation

**Fig. 1:**
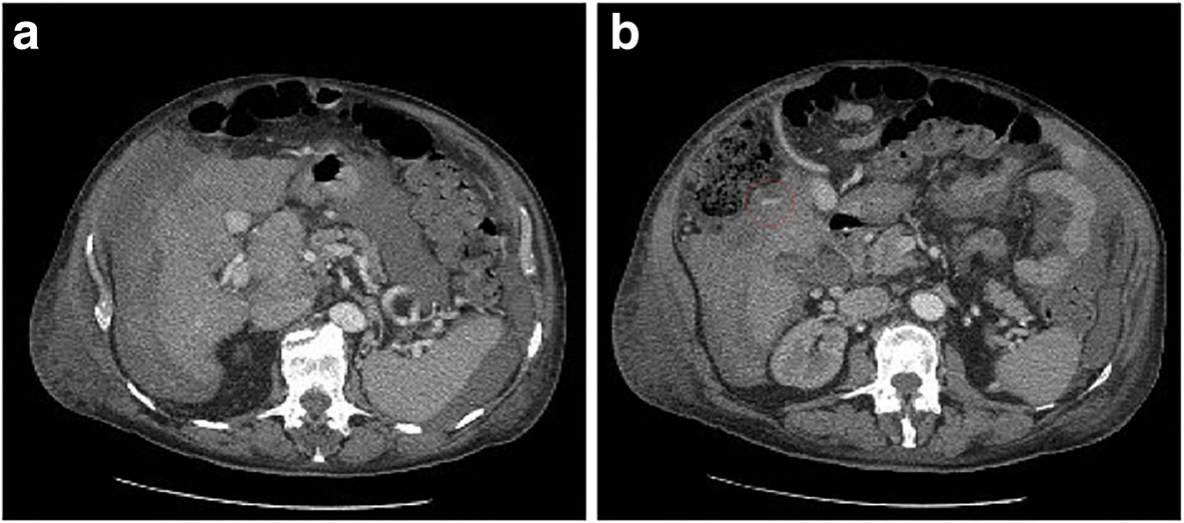
Abdominal computed tomography scan with intraperitoneal fluid with blood density (**a**), and evidence of the contrast leak (*white spread*) in proximity of the right lobe of the liver (**b**)

**Fig. 2:**
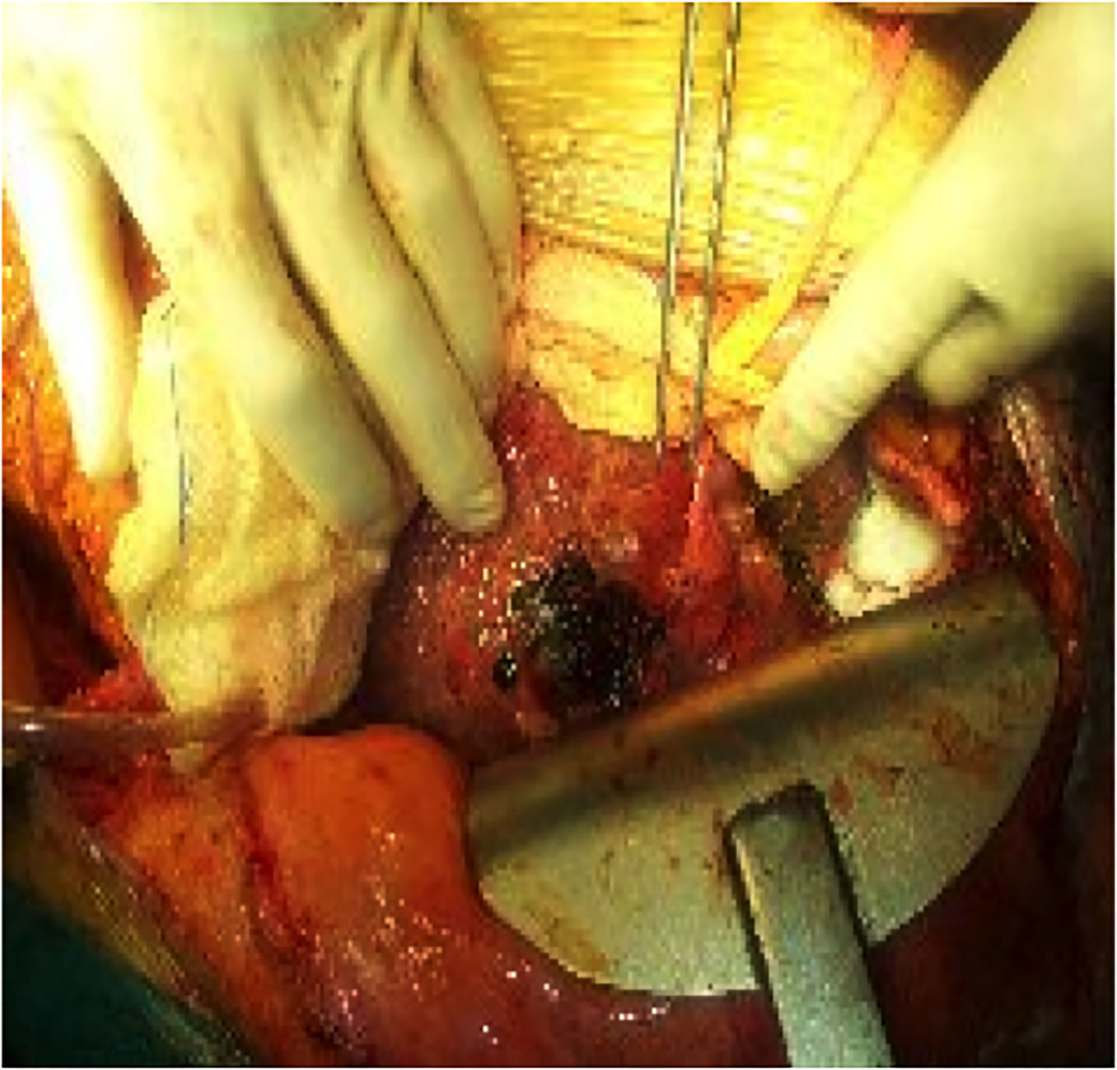
Final result of bleeding control from ruptured hepatocellular carcinoma using radiofrequency ablation

We present a case report of a 78-year-old white man with alcoholic cirrhosis and multifocal HCC with ascites and portosystemic encephalopathy. In his past history (2 years before) he had a wedge resection of segment II for HCC (G2). Since then he was followed-up annually, including a computed tomography (CT) scan, by our medical department. He was not considered for LT due his advanced age. He presented to our emergency room for ascitic decompensation with abdominal tension and lower limb edema. During his recovery, his hematocrit suddenly dropped (hemoglobin from 9.3 g/L to 6.7 g/L in 3 hours); an abdominal CT scan showed multiple and bilateral foci of HCC with evidence of acute bleeding from one of them (Fig. 1). His Model for End-Stage Liver Disease (MELD) score was 19; his Child–Pugh score was C11; total bilirubin was 8 mg/dl and alpha-fetoprotein (AFP) 604 ug/L. He was hemodynamically unstable and compromised so he was urgently transferred to our operating room (OR) for hemorrhagic shock. A middle line laparotomy was performed and a massive hemoperitoneum was found (4 L). His liver was cirrhotic with recanalization of umbilical vein and collateral vessels. His liver was completely subverted by a tumor and there was bleeding from two lacerations on segments II and IV, which was uncontrollable with conventional hemostatic techniques (argon beam, oxidized regenerated cellulose, and fibrin glue). Due to his condition, his poor liver function reserve, and the multifocal tumor it was decided to carry out the coagulation of the multiple vascular afferents of each single mass by RFA. Multiple RFA cycles were performed circumferentially on both nodules for a total of 40 minutes. Hemostasis was achieved; the RFA controlled the bleeding from our patient’s ruptured HCC (Fig. 2). Operation time was 90 minutes. During the operation he needed transfusions of three packed red blood cells (PRBC) transfusions and drug support with noradrenaline 0.4 gamma/Kg/minute and dopamine 2 gamma/kg/minute. He was transferred to our intensive care unit (ICU) for postoperative monitoring. On postoperative day (POD) 2 he was discharged and reassigned to our medical floor, without vasopressor therapy. His peak postoperative transaminase levels were aspartate aminotransferase (AST) 659 UI/L and alanine aminotransferase (ALT) 260 UI/L but he did not develop liver failure. The main problem was the hepatorenal syndrome that occurred on POD9 due to the progression of his underlying liver disease; he did not need renal replacement therapy. He was discharged from our medical department; his general condition was satisfactory. He was followed-up in our clinic by our palliative care team, but unfortunately he died 2 months later due to a progression of his disease.

## Discussion

Recent progress in diagnostics has facilitated early identification of HCC. Despite this advancement, some patients continue to be diagnosed at a late stage. Ruptured HCC often represents a more advanced tumor stage, as reflected by the tumor size, tumor number, vascular invasion, and tumor marker values. Furthermore, a comparison between ruptured HCC and non-ruptured HCC revealed that spontaneous tumor rupture was more frequent among patients with a poor liver functional reserve, as reflected by Child–Pugh grade and liver damage classification [2]. Diagnosing ruptured HCC can be difficult, although the development of imaging studies have improved the rate of preoperative diagnosis. An abdominal-pelvic CT scan is currently the most important radiological investigation to perform as the patient becomes hemodynamically stable [1]. The second step is the control of bleeding: whatever technique is chosen, the main goal is to obtain effective hemostasis and stabilize cardiorespiratory reserve. The tumor treatment is secondary: there is actually no evidence to recommend an invasive approach, such as emergency liver resections. Many scholars tend to choose emergency liver resection because it may effectively restore complete hemostasis and at the same time resect the primary lesions. However, even if the bleeding is stopped temporarily, death due to liver function failure and/or other comorbidity of the patient are important reasons to consider for patient outcomes [15]. Most patients lack the functional reserve to tolerate surgery and the disease is often surgically unresectable at the time of rupture. Hepatic reserve should be evaluated once hemostasis is achieved, and only when selected patients are in a stable phase should tumor treatment, such as elective hepatectomy or TAE, be performed [16]. TAE in stable patients with ruptured HCC and active bleeding is currently the treatment of choice, even if the optimal standards and therapeutic values of treatment have not reached a unified consensus. Its advantage lies in its simplicity, minimal invasiveness, fast hemostasis, and rapid postoperative recovery. TAE followed by elective hepatectomy is also considered an effective strategy for patients with ruptured HCC [4]. In selected patients, prolonged survival is also possible using TAE as initial therapy with or without a delayed resection and systemic therapy [17]. However, TAE is not always feasible without major procedural complication. A recent study evaluated the outcome of emergent embolization of ruptured HCC; patients with a Child–Pugh score of B/C, those with a MELD of 10 or more, and patients with portal vein thrombosis prior to embolization had a higher risk of death [18]. If embolization is unavailable and patients are hemodynamically unstable, then emergency surgery may be necessary. The goals are rapid and effective control of bleeding. The preferred technique of hemostasis is packing, after temporary clamping of the hepatic pedicle. The techniques of suture, intratumoral injection of alcohol [19], and ligation of the proper hepatic artery [5] should no longer be performed because of reports of low efficacy and high morbidity. Patients with a poor liver function reserve cannot tolerate surgical resection or aggressive angiographic intervention. Therefore, Child–Pugh and MELD, which reflect reserved hepatic function, could be important pretreatment factors. In some cases, conservative treatments are selected, but recent studies showed that this may not be the best approach. A study from Taiwan [20] concluded that a conservative approach was associated with a significantly worse survival rate compared with hepatectomy alone or in combination with TAE. Jin *et al*. [21] suggested that the post-treatment outcomes of surgery or TAE are better than those of supportive care in these patients, and that surgical hemostasis might provide better survival benefit than TAE. In this setting, tumor RFA is a possible therapeutic policy that has exhibited significant progress in the last decade. In recent years, the efficacy of this method for achieving hepatic hemostasis has become greatly appreciated in the treatment of liver trauma and in cases of ruptured liver tumors [1]. RFA is a minimally invasive treatment and can be performed laparoscopically or during open surgery in patients who are in poor general condition or who have liver dysfunction. RFA uses pulsed radiofrequency current to quickly heat and ablate large volumes of tissue. At temperatures above 60 °C, cellular proteins rapidly denature and coagulate. The ability of radiofrequency to coagulate tissues, arresting the microcirculating blood, is in fact responsible for its hemostatic effect [22]. Manikam *et al*. reported two cases of ruptured HCC in which RFA successfully achieved hemostasis [23]. Sun *et al*. performed RFA as both salvage therapy and curative treatment for spontaneous rupture of HCC [24]. Cheung *et al*. reported that the use of RFA for hemostasis during laparotomy greatly reduced the hospital mortality rate when compared with conventional hepatic artery ligation [14].

## Conclusions

In patients with spontaneous HCC rupture the first goal is hemostasis. To date there has been no prospective randomized controlled trial or well-designed comparative study designed to determine the best method of hemostasis. In hemodynamically unstable patients, or when embolization and/or resection are unavailable and/or unfeasible due to the patient’s condition, RFA is a good alternative or could be complementary to the classic emergency surgery. This study has shown that RFA is a safe and simple operative hemostasis method that can also be considered a procedure for life-threatening events in patients with end-stage liver disease. The treatment approach has to be chosen after evaluating liver reserve, liver tumor invasion, and life expectancy. A clear and comprehensive treatment protocol for these scenarios is essential.

## Abbreviations

AFP: Alpha-fetoprotein
ALT: Alanine aminotransferase
AST: Aspartate aminotransferase
CT: Computed tomography
HCC: Hepatocellular carcinoma
ICU: Intensive care unit
LT: Liver transplantation
MELD: Model for End-Stage Liver Disease
OR: Operating room
POD: Postoperative day
PRBC: Packed red blood cells
RFA: Radiofrequency ablation
SLB: Spontaneous liver bleeding
TAE: Transcatheter arterial embolization
